# Differences in the Prevalence of Fruit and Vegetable Consumption in Spanish Workers

**DOI:** 10.3390/nu12123848

**Published:** 2020-12-16

**Authors:** Elena Ronda-Pérez, Julia Campos-Mora, Alba de Juan, Teresa Gea, Alison Reid, Pablo Caballero

**Affiliations:** 1 Department of Community Nursing, Preventive Medicine and Public Health and History of Science, University of Alicante, 03690 Alicante, Spain; pablo.caballero@ua.es; 2 CIBER de Epidemiología y Salud Pública (CIBERESP), 28029 Madrid, Spain; 3 Preventive Medicine and Public Health Service, University Hospital of Alicante, 03010 Alicante, Spain; juliacamposmora@gmail.com (J.C.-M.); alba_djp@ua.es (A.d.J.); gea_mte@gva.es (T.G.); 4 School of Public Health, Curtin University, 6102 Perth, Australia; alison.reid@curtin.edu.au

**Keywords:** demographic characteristics, health behavior, education, fruit and vegetable consumption, work-related factors, occupation

## Abstract

The present study aims to examine the differences in daily fruit and vegetable consumption in the working population in Spain. A cross-sectional study was conducted, using data from the 2017 National Health Survey (*n* = 10,700 workers aged between 18 and 65 years). The daily consumption of fruit and vegetables was evaluated using two items included in a food frequency questionnaire. Occupations were classified into the 17 main groups of the National Classification of Occupations of 2011 (CNO-11). The prevalence (P) of daily fruit and vegetable consumption was calculated in relation to sociodemographic characteristics, health behaviors, work-related characteristics and occupations. Logistic regression analysis was performed to examine the association, with simple and adjusted Odds Ratio (aOR). The P of daily consumption of fruit and vegetables in workers was 60% for fruit and 40% for vegetables. After adjusting for sociodemographic characteristics and health behaviors, workers working night or rotating shifts had a lower consumption of fruits (aOR:0.9; *p* < 0.05), and those working on temporary contracts had a lower consumption of vegetables (aOR:0.8; *p* < 0.05). Engineers, scientists, health care workers and teachers had the highest fruit consumption (74.5%) and the highest vegetable consumption (55.1%). The lowest consumption of fruits was presented by the military (42.3%) and unskilled workers in the service sector (45.8%), and the lowest consumption of vegetables was presented by skilled construction workers (25.5%). These findings could aid in workplace health promotion and could be used in future studies to evaluate the impact of the activities adopted.

## 1. Introduction

Lifestyles are important population determinants of health. They are also risk factors related to the development of certain illnesses such as obesity, hypertension, dyslipidemia, diabetes, cardiovascular diseases and certain types of cancer [1,2]. Additionally, factors such as high alcohol consumption, tobacco use, unhealthy diet and lack of physical activity are related to occupation [3].

Unhealthy diets are characterized by low or lack of daily consumption of fruit and vegetables. According to the World Health Organization (WHO), fruit and vegetables are an essential part of a healthy diet. The WHO estimates that 1.7 million lives could be saved each year if fruit and vegetable consumption increased. It is recommended that people eat at least 400 g or five portions of fruit and vegetables each day in order to reduce the risk of developing communicable diseases and to help guarantee sufficient levels of dietary fiber [4].

Occupation is a dimension of one’s socioeconomic position that brings together lifestyles and living conditions related to one’s level of education and income. In general, a higher level of professional qualification brings with it employment with better working conditions, such as greater autonomy in the workplace and lower exposure to work-related risks, as well as a higher salary. Education provides people with a series of social and psychological resources that promote healthy lifestyles. In addition, higher incomes come with greater ability to satisfy basic human needs [5]. In this sense, various studies have shown that belonging to a higher socioeconomic class is related to the consumption of a Mediterranean Diet. The Mediterranean Diet is considered the paradigm of a balanced diet and is characterized by a high consumption of fresh fruits and vegetables, whole grains, fish, lean meats and low-fat dairy products [6,7].

One’s occupation also implies exposure to different working conditions which can be associated with poor dietary habits. For example, work-related stress, shift work, occupations with long working hours and frequent changes in the workplace are associated with low consumption of fruit and vegetables [8,9]. Factors that facilitate access to an unhealthy diet in the workplace have been shown to be important [10], perhaps because the best possible option is often fast food, or because the rhythm of work limits the time available for eating and/or choosing healthy foods [11]. Long working hours are also an additional working condition that has been associated with unhealthy dietary habits, such as skipping meals or consuming fast food, because of the lack of time for preparing meals [12].

Despite the differences that exist in working conditions, there are few studies that analyze the dietary differences between different occupations. Some of these studies have shown that there are differences in dietary habits between occupational groups, however, these studies have focused on categories such as “manual workers” [13]. Others have focused on specific workers such as those who work night shifts, such as nurses [14], bus drivers [15], airline workers [16] and industrial workers [17]. A study by Tanaka et al. showed that there were considerable differences in food consumption between different occupations in Japan [9]. Studies in Spain about dietary patterns in the working population are scarce. Some focused on specific occupations, such as university workers, and showed an insufficient consumption of fruits (only a third consumed three or more pieces of fruit a day) and vegetables (only half consumed two or more servings a day) [18]. Other studies compared dietary consumption between specific groups of workers, such as migrants and the Spanish-born, and showed that employment conditions had a limited influence on the lower consumption of fruit and vegetables among the migrant workers [19]. Another study examining the general population observed that groups with the lowest educational levels consumed lower quantities of vegetables [20]. This analysis is important because, from an occupational health perspective, companies play an important role in developing and integrating health promotion activities in the workplace [21,22]. A modifiable risk factor associated with greater mortality and disability is the low consumption of fruit and vegetables [23], and interventions in this area in the workplace have been shown to be effective [24]. The objective of this study was to examine the differences in the daily consumption of fruit and vegetables in the working population in Spain using data from the National Health Survey of 2017.

## 2. Materials and Methods 

### 2.1. Study Design and Sample

This was a cross-sectional study carried out using data from the Spanish National Health Survey (SNHS) of 2017 obtained from the National Statistics Institute (NSI). The SNHS is a representative sample of the non-institutionalized population residing in Spain, and the survey is carried out every five years with a target population of people who live in family households within the national territory. Information was collected throughout the year, from October 2016 to October 2017. To meet the survey’s objectives of providing estimates at the national and regional level, a theoretical sample of approximately 37,500 dwellings in 2500 census tracts were selected. Excluding empty and non-residential dwellings, the eligible sample consisted of 32,783 dwellings containing 33,046 households. Of those, 12% contained more than one household per dwelling and 14% had absent householders and so were excluded, leaving 23,457 eligible households. The final sample consisted of 23,090 households who participated in the survey (95%), 317 who refused (1%), 915 (4%) who were absent and 13 who were incapacitated. The data were collected through computer assisted telephone interviews. All individuals voluntarily participated in the SNHS and provided their informed consent. The researchers worked with a spreadsheet using anonymized data [25].

For the purposes of this study, we restricted the analysis to the 10,700 adults aged between 18 and 65 years that were employed at the time of the survey. The National Health Survey considers people to be working when, at the time of the interview, they have a contractual relationship through which they are paid in cash or in kind, are working for themselves, or are members of and work for production cooperatives.

### 2.2. Variables

The study variables, daily consumption of fruit and vegetables, were collected by the 2017 SNHS. Fruit included both fresh and frozen fruit, fruit conserves and dried fruit, but not fruit juice. Vegetables excluded potatoes and vegetable juices. Fruit juice, potatoes and vegetable juices were collected in the questionnaire but were not included in this study. Fruit and vegetable consumption were recorded as: 1 or more times per day; 4 to 6 times per week; 3 times per week; 1 or 2 times per week; less than once per week; or never. For the present study, an affirmative response to “consumption one or more times per day” was used to signify daily consumption.

Occupation was used as the explanatory variable. The NSI registered the occupation of individuals in the SNHS questionnaire using the National Classification of Occupations of 2011 (CNO-11) using a three-digit code (170 subgroups). The present study used the 17 principal groups of the CNO-11 [26,27].

Risky health behavior variables examined included tobacco consumption. Smokers were considered to be those who smoked (both daily or less than daily), ex-smokers were those who did not smoke at the time of the interview but had smoked in the past, and non-smokers were those who had never smoked. For physical activity, the variable “frequency of physical activity during free time” was used. This variable was derived from the question: Which of the scenarios listed below best describes how often you are physically active in your free time? People who are sedentary or physically inactive are people who have not engaged in exercise (“I don’t exercise. I occupy my free time almost completely with sedentary activities: reading, watching TV, going to the movies, etc.”). People who exercise occasionally are those who occasionally walk, cycle, garden, undertake light gymnastics and recreational activities that require light effort, etc. People considered to be active are those who engage in physical activity during their free time at various times during the month or various times during the week (sports, gymnastics, running, swimming, cycling, team games, etc.) [28]. For alcohol use, the variable “average daily alcohol consumption in a typical week during the last 12 months of normal activity” was used. The SNHS provided individualized self-reported data on the quantity and frequency of alcoholic beverages consumed in the last 12 months. Workers provided information on the usual frequency and quantity of beer, wine and spirits consumed during the preceding year by answering two questions. The first question asked: “How often do you usually consume alcoholic beverages?”. Possible responses for beer, wine and spirits were: (a) 3–4 times a day; (b) twice a day; (c) once a day; (d) 5–6 times a week; (e) 3–4 times a week; (f) once–twice a week; (g) 2–3 times a month; (h) once a month; (i) less than once a month but more than once a year; and, (k) never. The second question asked: “How many glasses or drinks do you usually have at any one time?”. Total alcohol consumption was taken as the sum of the values for the three types of beverage, assuming that: a 250 mL glass of beer contained 8 g of alcohol; a 120 mL glass of wine, 11.5 g of alcohol; and a typical “drink” of spirits, 16 g of alcohol. Alcohol intake was expressed in units of drink containing 10 g of alcohol. This variable was calculated in grams of pure alcohol by the SNHS [25]. Excessive consumption was considered to be ≥40 g daily for men and ≥20 g for women, the remaining values were considered non or low consumption [24].

The work characteristics examined were: type of contract (divided into three categories: fixed/indefinite; temporary; self-employed), weekly hours of work (divided into two categories: full time (defined as having worked >35 h per week) or part time (defined as ≤35 h per week)), type of workday (divided into two categories: fixed workday between 7 am and 8 pm; night shifts or rotating shifts), and level of stress, which was collected using the question “globally and taking into account your working conditions, how stressful do you consider your work to be, on a scale of 1 (not stressful at all) to 7 (very stressful) (recategorized for the present study into three categories: low: 1–2, medium: 3–5 and high: 6–7).

The sociodemographic characteristics included were: gender (male or female), age (measured quantitatively, divided into three age groups: 18–39, 40–49, 50–65) and educational level (based on the highest degree achieved and grouped into four categories: no studies or primary studies, secondary studies, high school, university studies).

### 2.3. Statistical Analysis

Firstly, we calculated the prevalence of the daily consumption of fruit and vegetables among Spanish workers by demographic characteristics, by health behavior and by occupation. To analyze the influence of occupation and the other covariables on fruit and vegetable consumption, odds ratios (OR) and adjusted odds ratios (aOR) were calculated, first with sociodemographic covariables and health risk behaviors and later for work characteristics. The aOR were calculated using logistic regression analysis with forward steps. The aOR were calculated using logistic regression analysis with forward steps, with an entrance and exit significance of 0.05 and 0.10, respectively. In the forward steps approach, the sociodemographic and health behavior variables are introduced in the model step by step, with the first variable being the most significant, provided that the significance is less than 0.05. Then, other variables are introduced one by one. However, if at any time a variable already introduced ceases to be significant (*p* > 0.10), the variable is extracted from the model. Confidence intervals were calculated at 95% (95% CI). For the analysis, the statistical package SPSS v.24 (IBM Corp., Armonk, NY, USA) for Windows was used.

## 3. Results

### 3.1. Daily Consumption of Fruit and Vegetables by Sociodemographic Characteristics and Health Behaviors

Table 1 presents the sociodemographic characteristics of the sample of 10,700 people (5639 men and 5061 women). The total prevalence of daily consumption of fruit and vegetables among workers was 60 percent for fruit and 40 percent for vegetables. As shown in the table, for both types of foods, men, younger workers aged 18–39, and those without studies or with a primary education level had a lower prevalence of daily fruit and vegetable consumption. The highest prevalence of consumption of fruit was found among those with a university degree (71%), and the lowest was among those under age 40 (52.4%). In the case of vegetables, the highest prevalence was among women (49.6%), and the lowest was among workers with low education levels (34.8%).

Smokers had a lower prevalence of daily consumption of fruits (49.7%) and vegetables (35.8%) compared with ex-smokers and non-smokers. Additionally, workers who were sedentary or those who consumed excess amounts of alcohol had a lower prevalence of daily consumption of fruit and vegetables in comparison with more active workers, non-drinkers and those with low alcohol consumption.

For both fruit and vegetables, the association between daily consumption by demographic characteristics and health behaviors was statistically significant for all of the variables analyzed, except in terms of vegetable consumption in workers who consumed alcohol excessively. For this group, no statistically significant differences were found.

### 3.2. Daily Consumption of Fruit and Vegetables by Work-Related Characteristics

Table 2 presents the differences in the consumption of fruit and vegetables by work-related characteristics. The lowest prevalence in terms of fruit consumption was observed among workers with temporary contracts (55.2%), among those working more than 35 h per week (61.8%), among those working night shifts or rotating shifts (57.7%) and those with high levels of stress (60.7%). The associations remained after adjusting for sociodemographic characteristics and risky health behaviors. Only the type of workday showed a statistically significant association, and workers with night shifts or rotating shifts had a lower consumption (OR 0.9; 95% CI: 0.8, 0.9).

Similar to fruit consumption, for vegetable consumption the lowest prevalences were observed in workers with temporary contracts (34.5%), in those working more than 35 h per week (40.3%) and in workers with night shifts or rotating shifts (39.6%). Vegetable consumption decreased with stress level, and workers with the lowest stress levels were those with lower consumption (38.9%). After adjusting for sociodemographic characteristics and health behaviors, only the type of contract, specifically workers with temporary contracts, showed a statistically significant association. These workers presented lower OR for consumption (OR 0.8; 95% CI: 0.7, 0.9).

### 3.3. Fruit and Vegetable Consumption by Occupation 

Engineers, science, health and education professionals (74.5%); other technicians, scientists and intellectuals (71.4%) and office workers who do not attend to the public (68.2%) were the occupations that had the greatest prevalence of daily fruit consumption. Compared with all other occupations and after adjusting for sociodemographic variables, differences remained for the three groups (OR 1.9; 95% CI: 1.6, 2.2), (OR 1.6; 95% CI: 1.4, 1.8) and (OR 1.3; 95% CI: 1.1, 1.6), respectively. However, after adjusting for both socioeconomic variables and health behaviors, and also for work-related characteristics, these differences disappeared and did not retain statistical significance (Table 3).

Military personnel (42.3%), agricultural workers, fishermen, construction workers, those working in the manufacturing or transportation industries (45.8%) and restaurant and commercial services workers (51.4%) had the lowest prevalence of daily fruit consumption. Compared with all other occupations and adjusting for sociodemographic variables, statistically significant associations remained for military personnel (OR 0.5; 95% CI: 0.3, 0.8), agricultural workers, fishermen, construction workers, those working in manufacturing or transportation (OR 0.8; 95% CI: 0.6, 0.7), and restaurant and commercial services workers (OR 0.7; 95% CI: 0.6, 0.8) (Table 3).

Engineers, science, health and education professionals were the only group that had a prevalence of daily vegetable consumption above 50 percent (55.1%). The difference between this group and all other occupations remained after adjusting for demographic variables, health behavior variables and job characteristics (OR 1.3; 95% CI: 1.2, 1.6).

Qualified construction workers, except machine operators (25.5%), agricultural workers, fishermen, qualified construction workers and those working in the manufacturing and transportation sectors (30.5%) and qualified workers in the agriculture, ranching, forestry and fishing sectors (31.5%) had the lowest prevalence of daily vegetable consumption. After adjusting for sociodemographic characteristics, health behaviors and work-related characteristics, only one occupation persisted in having a statistically significant low prevalence of daily vegetable consumption: qualified construction workers, except machine operators (OR 0.7; 95% CI: 0.6, 0.9) (Table 4).

Both in terms of fruit and vegetable consumption, once work-related characteristics were included in the model, there was no observed variation for any of the included occupations in any of the models.

## 4. Discussion

This study examined a representative sample of the Spanish working population and identified occupations associated with greater or lesser daily consumption of fruit and vegetables. The influence of sex, education level and age as well as the relevance of other health risks, such as tobacco use, exercise and alcohol consumption on daily fruit and vegetable consumption was also shown. In terms of working conditions, only the type of workday (for fruit consumption) and the type of contract (for vegetable consumption) were statistically significant predictors for low fruit or vegetable consumption.

Many studies that analyze population fruit and vegetable consumption from the perspective of socioeconomic differences do so using indicators of education, occupation and income [6,29]. Although all three are associated with fruit and vegetable consumption, the relationship is best explained and predicted by education level [6,7,30,31]. In general, individuals with lower socioeconomic status generally have a lower consumption of fruit and vegetables [7,32] compared with individuals of higher socioeconomic status. In this study, we also observed a positive association between education level and daily consumption. This could be explained by the fact that a higher level of education brings with it greater knowledge of nutrition and the benefits of a balanced diet [6,33].

Occupation implies exposure to different working conditions that have been associated with poor nutrition. This study found an association between the type of workday and fruit consumption in Spanish workers. This finding coincides with what other studies have found and provides evidence that workers with night shifts or rotating shifts consume less fruit and vegetables [15,16]. This has been explained, in part, by the fact that preparing a healthy diet based on greater quantities of fruits and vegetables requires more time, and as such, types of workdays with flexible hours facilitate this type of consumption [29]. In this current study, we found different consumption patterns of fruit and vegetables. Fruit tends to be sweet and soft, is often eaten raw and is frequently considered a snack or dessert and so can be more easily consumed. In contrast, vegetables have a harder texture, generally require cooking and are more often eaten as part of a meal [34]. Furthermore, this study excluded the consumption of potato from the analysis, and for many participants this may have been the only vegetable consumed, thus underestimating vegetable consumption.

Other working conditions, such as the level of stress or availability of cafeterias or restaurants that provide access to fruit and vegetables, also influence workers’ nutrition. Raulio et al. [35] observed that workers with rotating shifts and those with high levels of stress consume more processed food and vending machine foods and eat less frequently in cafeterias. In our study, we observed similar results in terms of fruit consumption: there was lower consumption with higher levels of stress. However, it is worth noting that in terms of the relationship to vegetable consumption, there was a greater consumption associated with a higher level of stress.

Our results show that manual workers from sectors such as agriculture, manufacturing and construction had a lower consumption of fruit compared to non-manual workers such as engineers and mid-level technicians and administrators, where consumption was greater. Earlier studies consistently support this finding and confirm that manual workers consume less fruit and vegetables compared to all other occupational categories [9,33,36,37]. The results are similar in terms of vegetables, where a manual profession, such as construction work, has lower consumption, with greater consumption among scientific professionals and engineers. Socioeconomic status can partly explain these findings, given that, in general, manual workers tend to have a lower level of education [38]. In our study, other occupations in the service sector identified as having low consumption, including transport workers and restaurant and commercial services workers. Different studies show that these workers tend to more frequently consume processed foods and less fruit and vegetables [35,39].

Another possible explanation for the lower fruit and vegetable consumption is that manual occupations or those that require fewer qualifications tend to work longer workdays, have more shift work and have higher levels of stress, which have been associated with lower daily consumption of fruit and vegetables [40,41]. It is interesting to note that there was low daily consumption of fruit among military personnel. This finding has been described in earlier studies, which found a high prevalence of overweight people in this group, in addition to an insufficient consumption of fruit and vegetables and a high consumption of sugary drinks [42].

Other factors have been shown to be related to the low consumption of fruit and vegetables, such as gender, age and health behaviors. According to earlier studies, men have lower levels of consumption of fruit and vegetables compared with women [43]. This finding suggests that there could be gender differences with respect to awareness about health in general and the importance of following a healthy diet in particular. Age is another sociodemographic characteristic that has shown a statistically significant association with fruit and vegetable consumption. The greater the age, the greater the consumption. These results agree with earlier studies that put forward that young people are more vulnerable to unhealthy behaviors, they skip primary meals, consume more processed foods and also place lower importance on healthy habits [6,44].

This study includes limitations that should be considered when interpreting its results. Firstly, food consumption data were not obtained using an objective measure such as a registration system. Food consumption was self-reported. However, food questionnaires are the method that is most often used to evaluate food consumption in observational studies due to their simplicity and low cost [45]. Secondly, we used only two items of a food frequency questionnaire to measure fruit and vegetable intake. Other work has shown the validity of this method [46]; however, we acknowledge that including information about other foods consumed would have contributed to a better understanding of the observations observed in this study. Thirdly, the survey did not collect information about the quantity of consumption, which is necessary to determine if the respondents were meeting dietary guidelines for fruit and vegetable consumption. A strength of this study is the fact that findings were obtained from a sample of the Spanish population that is representative at the national level and includes Spanish workers from all of the occupational categories. Additionally, a reference group of employed persons was used to make comparisons instead of using the whole population, which allowed us to avoid the healthy worker bias that is produced in studies that compare those with and without employment [47].

## 5. Conclusions

After analyzing the variables related to nutritional practices concerning fruit and vegetable consumption, we observed the influence of joint exposure to factors related to socioeconomic status and lifestyles, as well as the importance of shift work. Future research should seek to identify factors that explain the observed differences in prevalence between occupations that go beyond working conditions. These findings can serve as a reference to support developing health promotion activities in companies and to evaluate the impact of the adopted activities.

## Figures and Tables

**Table 1:** Daily consumption of fruit and vegetables by sociodemographic characteristics and health behaviors.

	Sample	Fruit Consumption *n* (%)	OR (95% CI)	Vegetable Consumption *n* (%)	OR (95% CI)
By Sociodemographic Characteristics					
Gender					
Male	5639	3149 (55.8%)	0.6 * (0.5;0.6)	1850 (32.8%)	0.5 * (0.5;0.5)
Female	5061	3478 (68.7%)	Ref.	2512 (49.6%)	Ref.
Age					
18–39	3571	1871 (52.4%)	Ref.	1278 (35.8%)	Ref.
40–49	3518	2150 (61.1%)	1.4 (1.3;1.6)	1402 (39.9%)	1.2 (1.1;1.3)
50–65	3611	2606 (72.2%)	2.4 (2.1;2.6)	1683 (46.6%)	1.6 (1.4,1.7)
Education level					
No studies or primary studies	1044	588 (56.3%)	0.5 * (0.5;0.6)	363 (34.8%)	0.6 * (0.5;0.7)
Secondary studies	3667	2101 (57.3%)	0.5 * (0.5;0.6)	1336 (36.4%)	0.6 * (0.6;0.7)
High school	2864	1720 (60.1%)	0.6 * (0.6;0.7)	1176 (41.1%)	0.8 * (0.7;0.9)
University studies	3126	2219 (71.0%)	Ref.	1487 (47.6%)	Ref.
By Health Behavior					
Smoking status					
Smoker	3200	1589 (49.7%)	0.5 * (0.4; 0.5)	1144 (35.8%)	0.8 * (0.7; 0.8)
Ex-Smoker	2906	1957 (67.3%)	1.0 (0.9; 1.1)	1286 (44.3%)	1.1 (1.0;1.2)
Non-smoker	4585	3078 (67.1%)	Ref.	1933 (42.2%)	Ref.
Exercise					
Active	3291	2240 (68.1%)	Ref.	1440 (43.8%)	Ref.
Sedentary	7406	4386 (59.2%)	0.7 * (0.6;0.7)	2923 (39.5%)	0.8 * (0.8;0.9)
Alcohol Status					
Non or low consumption	10,449	6495 (62.2%)	Ref.	4263 (40.8%)	Ref.
Excessive consumption	252	133 (52.8%)	0.7 (0.5;0.9)	100 (39.7%)	1.0 (0.7;1.2)

OR: Odds Ratio, Ref. Reference group (Ref = 1). * *p*-Value < 0.05 for the OR.

**Table 2:** Daily consumption of fruit and vegetables by work-related characteristics.

**Fruits**	**Sample**	**Daily Consumption *n* (%)**	**OR** **(95% CI)**	**ORa1** **(95% CI)**	**ORa2** **(95% CI)**
Type of contract					
Fixed/indefinite	6966	4455 (64.0%)	Ref	Ref	Ref
Temporary	1839	1016 (55.2%)	0.7 * (0.6;0.8)	0.8 * (0.8;0.9)	0.9 (0.8;1.0)
Self-employed	1850	1133 (61.2%)	0.9 (0.8;1.0)	0.9 (0.8;1.0)	0.9 (0.8;1.0)
Weekly work hours					
≤35 h	1395	884 (63.4%)	Ref	Ref	Ref
>35 h	9250	5713 (61.8%)	0.9 (0.8;1.0)	1.0 (0.9;1.1)	1.0 (0.9;1.1)
Type of workday					
Fixed workday between 7am and 8pm	7713	4907 (63.6%)	Ref	Ref	Ref
Night shifts or rotating shifts	2933	1691 (57.7%)	0.8 * (0.7;0.8)	0.8 * (0.8;0.9)	0.9 * (0.8;0.9)
Stress level					
Low	3188	2010 (63.0%)	1.1 (1.0;1.2)	1.2 * (1.1;1.3)	1.2 (1.0;1.3)
Medium	4842	3000 (62.0%)	1.1 (1.0;1.2)	1.1 (1.0;1.2)	1.1 (1.0;1.2)
High	2603	1581 (60.7%)	Ref	Ref	Ref
**Vegetables**	**Sample**	**Daily Consumption *n* (%)**	**OR** **(95% CI)**	**ORa1** **(95% CI)**	**ORa2** **(95% CI)**
Type of contract					
Fixed/indefinite	6965	2955 (42.4%)	Ref	Ref	Ref
Temporary	1838	634 (34.5%)	0.7 * (0.6;0.8)	0.8 (0.7;0.9)	0.8 (0.7;0.9)
Self-employed	1850	753 (40.7%)	0.9 (0.8;1.0)	1.0 (0.9;1.1)	1.0 (0.9;1.1)
Weekly work hours					
≤35 h	1395	608 (43.6%)	Ref	Ref	Ref
>35 h	9249	3727 (40.3%)	0.9 (0.8;1.0)	1.0 (0.9;1.2)	1.0 (0.9;1.2)
Type of workday					
Fixed workday between 7am and 8pm	7713	3176 (41.2%)	Ref	Ref	Ref
Night shifts or rotating shifts	2934	1161 (39.6%)	0.9 (0.9;1.0)	1.0 (0.9;1.1)	1.0 (0.9;1.1)
Stress level					
Low	3188	1241 (38.9%)	0.8 * (0.7;0.9)	0.9 (0.8;1.0)	0.9 (0.8;1.0)
Medium	4843	1963 (40.5%)	0.9 (0.8;1.0)	0.9 (0.8;1.0)	0.9 (0.8;1.0)
High	2603	1132 (43.5%)	Ref	Ref	Ref

Ref. Reference group (Ref = 1). OR: Odds Ratio, ORa1: Adjusted Odds Ratios by demographic variables, ORa2: Adjusted Odds Ratios by sociodemographic and health behavior variables. * *p*-Value < 0.05 for the OR.

**Table 3:** Daily consumption of fruit by occupation.

	Size	Daily Consumption *n* (%)	OR ● (95% CI)	ORa1 ● (95% CI)	ORa2 ● (95% CI)
Occupation					
Management	325	203 (62.5%)	1.0 (0.8;1.3)	0.8 (0.6;1.0)	0.8 (0.6;1.0)
Engineers, science, health and education professionals	1047	780 (74.5%)	1.9 * (1.6;2.2)	1.1 (1.0;1.3)	1.1 (0.9;1.3)
Other technicians, scientist and intellectuals	933	666 (71.4%)	1.6 * (1.4;1.8)	1.2 (1.0;1.4)	1.2 (1.0;1.5)
Support Technicians	1144	729 (63.7%)	1.1 (1.0,1.2)	1.0 (0.9;1.1)	1.0 (0.9;1.1)
Office workers who do not attend to the public	466	318 (68.2%)	1.3 * (1.1;1.6)	1.2 (1.0;1.5)	1.2 (1.0;1.4)
Office workers who attend to the public	562	372 (66.2%)	1.2 (1.0;1.5)	1.1 (0.9;1.3)	1.1 (0.9;1.3)
Restaurant and commercial services workers	1436	738 (51.4%)	0.6 * (0.5;0.7)	0.7 * (0.6;0.8)	0.7 * (0.6;0.8)
Health care workers	750	508 (67.7%)	1.3 * (1.1;1.5)	1.1 (1.0;1.3)	1.1 (1.0;1.4)
Security services	255	151 (59.2%)	0.9 (0.7;1.1)	1.0 (0.8;1.3)	1.0 (0.8;1.3)
Qualified workers in the agricultural ranching, forestry and fishing sector	363	232 (63.9%)	1.1 (0.9;1.4)	1.3 (1.0;1.6)	1.3 (1.0;1.7)
Qualified construction workers, except machine operators	448	238 (53.1%)	0.7 * (0.6;0.8)	1.0 (0.8;1.3)	1.0 (0.8;1.3)
Skilled industry workers	716	394 (55.0%)	0.7 * (0.6;0.9)	1.0 (0.9;1.2)	1.0 (0.9;1.2)
Fixed machinery and plant operators and assemblers	337	201 (59.6%)	0.9 (0.7;1.1)	1.3 (1.0;1.7)	1.3 (1.0;1.7)
Drivers and mobile machinery	480	252 (52.5%)	0.7 * (0.6;0.8)	1.0 (0.8;1.2)	1.0 (0.8;1.2)
Unskilled workers in the service sector	883	593 (67.2%)	1.3 (1.1;1.5)	1.2 (1.0;1.4)	1.2 (1.0;1.4)
Agricultural workers, fishermen, construction workers, workers in manufacturing or transportation industries	485	222 (45.8%)	0.5 * (0.4;0.6)	0.8 * (0.6;0.7)	0.8 * (0.6;0.9)
Military	71	30 (42.3%)	0.5 * (0.3;0.7)	0.5 * (0.3;0.8)	0.5 * (0.3;0.8)

* *p*-Value < 0.05 OR, Odds Ratio, ORa1 Adjusted Odds Ratios by demographic variables and health behavior variables. ORa2 Adjusted Odds Ratios by demographic variables, health behavior variables and job characteristics. ● All the other groups are the reference group.

**Table 4:** Daily consumption of vegetables by occupation.

	Size	Daily Consumption *n* (%)	OR ● (95% CI)	ORa1 ● (95% CI)	ORa2 ● (95% CI)
Occupation					
Management	325	155 (47.7%)	1.3 * (1.1;1.7)	1.2 (1.0;1.6)	1.2 (1.0;1.5)
Engineers, science, health and education professionals	1047	577 (55.1%)	1.9 * (1.7;2.2)	1.3 * (1.2;1.6)	1.3 * (1.2;1.6)
Other technicians, scientist and intellectuals	933	415 (44.5%)	1.2 (1.0;1.4)	1.0 (0.9;1.2)	1.0 (0.9;1.2)
Support Technicians	1145	467 (40.8%)	1.0 (0.9;1.1)	0.9 (0.8;1.1)	0.9 (0.8;1.1)
Office workers who do not attend to the public	465	204 (43.9%)	1.1 (0.9;1.4)	1.0 (0.8;1.2)	1.0 (0.8;1.2)
Office workers who attend to the public	562	226 (40.2%)	1.0 (0.8;1.1)	0.8 (0.7;1.0)	0.8 (0.7;1.0)
Restaurant and commercial services workers	1435	520 (36.2%)	0.8 * (0.7;0.9)	0.8 * (0.7;0.9)	0.8 (0.7;1.0)
Health care workers	751	369 (49.1%)	1.4 * (1.2;1.7)	1.2 * (1.0;1.4)	1.2 * (1.1;1.4)
Security services	254	90 (35.4%)	0.8 (0.6;1.0)	1.0 (0.8;1.3)	1.0 (0.8;1.3)
Qualified workers in the agricultural ranching, forestry and fishing sector	362	114 (31.5%)	0.7 * (0.5;0.8)	0.8 (0.6;1.0)	0.8 (0.6;1.0)
Qualified construction workers, except machine operators	447	114 (25.5%)	0.5 * (0.4;0.6)	0.7 * (0.6;0.9)	0.7 * (0.6;0.9)
Skilled industry workers	715	228 (31.9%)	0.7 * (0.6;0.8)	0.9 (0.8;1.1)	0.9 (0.8;1.1)
Fixed machinery and plant operators and assemblers	336	147 (43.8%)	1.1 (0.9,1.4)	1.6 * (1.2;2.0)	1.6 * (1.3;2.0)
Drivers and mobile machinery	480	171 (35.6%)	0.8 (0.7;1.0)	1.3 (1.0;1.5)	1.3 (1.0;1.5)
Unskilled workers in the service sector	883	392 (44.4%)	1.2 (1.0;1.4)	1.0 (0.9;1.2)	1.0 (0.9;1.2)
Agricultural workers, fishermen, construction workers, workers in manufacturing or transportation industries	485	148 (30.5%)	0.6 * (0.5;0.8)	0.9 (0.7;1.1)	1.0 (0.8;1.2)
Military	71	23 (32.4%)	0.7 (0.4;1.2)	0.9 (0.5;1.5)	0.9 (0.5;1.5)

* *p*-Value < 0.05 OR, Odds Ratio, ORa1 Adjusted Odds Ratios by demographic variables and health behavior variables. ORa2 Adjusted Odds Ratios by demographic variables, health behavior variables and job characteristics. ● All the other groups are the reference group.
